# Incorporating natural ecosystems into global health and food security programmes

**DOI:** 10.2471/BLT.20.252098

**Published:** 2020-07-08

**Authors:** Anila Jacob, Kiersten Johnson, Robert Cohen, Sara E Carlson

**Affiliations:** a Smithsonian Institution, 600 Maryland Avenue SW, Washington, DC, 20002, United States of America (USA).; b Bureau for Resilience and Food Security, United States Agency for International Development, Washington, DC, USA.; c Camris International, MD, USA.; d Bureau for Economic Growth, Education and Environment, United States Agency for International Development, Washington, DC, USA.

Natural ecosystems such as forests, coral reefs, mangroves and grasslands provide essential goods and services that support human health and food security. Examples of these ecosystem services include crop pollination by wild pollinators, regulation of water flow by mangroves and provision of wild foods and natural medicines by forests. Globally, ecosystem degradation due to deforestation, overharvesting, pollution, poorly planned infrastructure projects, climate change, urbanization and other threats compromises ecosystem function and disrupts the flow of ecosystem services.

The Rockefeller Foundation-*Lancet* Commission on Planetary Health noted that negative environmental trends pose serious threats to the global health gains of the past decades.^1^ The Commission highlights environmental degradation as a major issue that could reverse many of the development advances made in recent decades such as increased life expectancy, decreased child mortality and lower rates of extreme poverty. Mechanisms through which global environmental change affects human health and food security include variations in the availability and quality of food, water and natural medicines; changes in the exposure to zoonotic, vector-borne and other infectious pathogens, as illustrated by the coronavirus disease 2019 (COVID-19) pandemic, as well as alterations in the frequency and intensity of extreme weather events.

Incorporating natural ecosystems into global health and food security programmes can help meet the goals of the health and food security sectors while contributing to the improved management and sustainability of ecosystems and the important services they provide. Here we suggest three practical ways in which natural ecosystems can be incorporated into global health and food security programmes to achieve these goals more sustainably and ensure that ecosystem degradation does not undermine previous achievements in these sectors. These suggestions will be most useful for international development donors, programme managers and technical specialists who fund and implement global health and food security programmes. We link each suggestion with a summary of the relevant evidence that supports it and specific types of actions to implement the suggestions.

First, ecosystem-related questions should be included in household surveys, as appropriate. Many global health and food security programmes conduct household surveys throughout project implementation. Incorporating relevant survey questions can help advance understanding of the ecosystem goods and services that target households rely on, of perceived changes to the availability of these goods and services, and of how household reliance on local ecosystems varies based on socioeconomic status, gender and other factors, among other topics.

A growing body of research demonstrates the many different ways that households benefit from natural ecosystems. For example, an analysis conducted in 2016 involving 7975 households in 24 tropical countries found that over three-quarters of study households harvested wild foods from local ecosystems for subsistence and sale.^2^ Wild foods are often rich in micronutrients, supplement carbohydrate-rich diets common in many low-income countries, and can be an important source of income for poorer households and those experiencing shocks.^2^^,^^3^ A global analysis found that many foods like pumpkin, mango and melon that are rich in micronutrients including vitamin A, iron and folic acid require animal pollinators for production.^4^ Natural ecosystems also provide medicinal plants for use in traditional medicine; an estimated 80% of people in low-income countries rely on traditional medicine to treat various ailments such as infections, inflammation and cardiovascular disease.^5^

Examples of ecosystem-related survey questions that reflect the current state of the evidence could focus on household use of wild foods such as fish, insects, meat, fruits and vegetables; use of wild foods during times of shock or crisis; and changes in the availability of wild foods due to local environmental change. Other potential topics to explore through survey questions include the status of local pollinator populations on household farms and the effects of local environmental change on crop productivity and the availability of medicinal plants. These types of questions, incorporated within the surveys and tailored to the local context, provide valuable information that can help guide activities aimed at sustaining the natural resource base and its contributions to health and food security.

Second, efforts to improve ecosystem management should be supported. The global health and food security sectors can support a range of activities that strengthen the natural resource base critical to optimizing the effectiveness and sustainability of their programmes. Scientific consensus that the earth’s natural ecosystems have entered an unprecedented era of degradation, raising concerns about their ability to continue providing ecosystem services, is increasing.

A growing body of evidence demonstrates linkages between natural ecosystems and global health and food security priorities, such as decreasing childhood illness and improving food security. For instance, a recent analysis found that higher upstream tree cover was associated with lower probability of diarrheal disease among children from rural households living in downstream communities in 35 low- and middle-income countries in Africa, Asia, the Caribbean, Europe and Latin America.^6^ Mechanisms to explain how tree cover can influence the risk of diarrheal disease include filtration of pollutants and displacement of human activities that pollute watersheds.^6^ Another study found that children in 27 low- and middle-income countries living within 3 km of forests had 25% greater dietary diversity, including increased consumption of vitamin A-rich fruits and vegetables, compared to children living farther away from forests.^3^ Access to forests can influence diets through the availability of forest products for direct consumption or to sell to purchase food. Forests also provide habitat for wild pollinators, which can enhance the productivity of nutritious foods including fruits and vegetables.^3^

Research also illustrates how ecosystem degradation can impair progress towards global health and food security objectives. In an analysis of 15 sub-Saharan African countries, deforestation in West Africa was negatively associated with dietary diversity in young children and recent consumption of nutritious foods including legumes, nuts and vitamin A-rich fruits and vegetables.^7^ Declines in wild fisheries productivity could result in at least 10% of the global population facing deficiencies of micronutrients like zinc, iron and vitamin A, as well as polyunsaturated fatty acids.^8^ A 50% loss of pollination services could lead to significant decreases in global fruit, vegetable, nut and seed production and be associated with 700 000 additional deaths and 13.2 million disability-adjusted life years annually worldwide.^9^

Actions that the global health and food security sectors can take to improve ecosystem management include promoting policies that protect ecosystems, communicating the importance of ecosystem stewardship to programme beneficiaries and stakeholders, collaborating with environmental organizations that work on ecosystem management in their target geographies, and advocating for increased funding for conservation programmes that may benefit people and natural systems. As natural ecosystem degradation has traditionally been seen as an environmental issue, global health and food security practitioners can be important advocates for elevating ecosystem considerations as a priority development issue with direct bearing on the success of their programmes.

Third, potential negative effects of global health and food security programmes on ecosystems should be minimized, and environmentally friendly practices should be maximized. Global health and food security programmes may have unintended negative consequences on natural ecosystems, which in turn can compromise the provision of critical ecosystem services. Conversely, promoting environmentally friendly programme approaches, whenever feasible, can minimize effects on the ecosystem while meeting programme objectives. When a more environmentally friendly alternative approach is not an option, programmes can undertake other types of efforts to minimize ecosystem damage, such as educational campaigns.

Agricultural expansion into biologically sensitive areas, crop monocultures with intensive use of pesticides and fertilizers, and concentrated livestock production are significant threats to natural ecosystems. These threats can lead to ecosystem degradation through habitat loss, pollution and soil degradation, with the loss of important ecosystem services, such as the provision of water and wild foods, pollination and soil nutrient cycling.^10^ In the global health sector, misuse of malaria bed nets can be harmful to aquatic ecosystems. In some areas, local communities use these bed nets for fishing, which has led to reduced bed net coverage for malaria prevention and the capture of juvenile fish in nursery grounds, with subsequent decreases in fishery productivity.^11^

More environmentally friendly agricultural practices like agroforestry, intercropping, natural pest control and mixed crop and livestock farming can decrease effects on the ecosystem while meeting food security goals.^12^ In the case of malaria bed net misuse, educational programmes that accompany distribution campaigns and close follow-up with target communities can encourage proper use.^11^ In some cases, consulting with environmental organizations can help global health and food security programmes identify and implement ecologically sensitive activities that meet their goals while minimizing ecosystem impacts.

Natural ecosystems and the services they provide are an essential foundation for human health and food security. The convergence of multiple threats has led to widespread environmental degradation and disruptions in ecosystem functionality worldwide, with potential negative effects on the provision of critically important ecosystem services. Our suggestions provide concrete ways in which the global health and food security sectors can engage in environmental stewardship to meet their goals more sustainably and ensure that ecosystem degradation does not undermine their progress.

When implementing these suggestions, we understand that there are trade-offs that programmes must consider, related to costs, time, available resources and other factors. We acknowledge these trade-offs but contend that the weight of the evidence supports the incorporation of ecosystem considerations whenever feasible. The rate of ecosystem degradation globally and the potential implications for human health and food security warrant engagement and comprehensive, wide-ranging approaches to reverse current environmental trends.
